# Text Message Feedback to Support Mindfulness Practice in People With Depressive Symptoms: A Pilot Randomized Controlled Trial

**DOI:** 10.2196/mhealth.7095

**Published:** 2017-05-02

**Authors:** Susanne Kraft, Markus Wolf, Thomas Klein, Thomas Becker, Stephanie Bauer, Bernd Puschner

**Affiliations:** ^1^ Section Process-Outcome Research Department of Psychiatry II Ulm University Günzburg Germany; ^2^ Clinical Psychology and Psychotherapy Research Institute of Psychology University of Zurich Zurich Switzerland; ^3^ Center for Psychotherapy Research University Hospital Heidelberg Heidelberg University Heidelberg Germany

**Keywords:** mindfulness, text messaging, pilot study, randomized controlled trial

## Abstract

**Background:**

It has been shown that mindfulness practice can be helpful in preventing relapse from depression. However, practicing mindfulness regularly at home is often a challenge for people with depression. Mobile phone text messaging (short message service, SMS) may be a feasible approach to assist regular mindfulness home practice.

**Objective:**

The aim of this study was to evaluate the feasibility of text message–based feedback to support mindfulness practice in people with depressive symptoms after inpatient psychiatric treatment.

**Methods:**

Participants received a manualized group introduction to three mindfulness exercises during inpatient treatment and were randomized at hospital discharge. All participants were asked to practice the exercises daily during the 4-month follow-up period. Only participants allocated to the intervention group received reinforcing feedback via mobile phone text messages after reporting their mindfulness practice via text message. Participation rates and satisfaction with the interventions were evaluated, and effects on relevant outcomes were explored.

**Results:**

Of the 176 eligible inpatients invited to participate, 65.9% (116/176) attended the introductory mindfulness group at least once, 33.0% (58/176) were willing to participate in the study, and 41 were randomized. The majority 85% (35/41) of these participants completed the study. Among the participants allocated to the intervention group (n=21), 81% (17/21) used the text message support at least once. The average number of text messages sent during the intervention period was 14 (SD 21, range 0-91). Satisfaction rates were high. Preliminary analyses of the effects of the intervention yielded mixed results.

**Conclusions:**

Findings indicate that text messaging following inpatient treatment is feasible for some, but not for all people with depressive symptoms. Modest use of the text messaging intervention and its mixed effects imply that dose and ingredients of the intervention should be increased for this group of patients in a future full-size RCT. Such a larger study should also include a process evaluation to investigate moderators of the effect of mindfulness practice and text message feedback on clinical outcome.

**Trial Registration:**

International Standard Randomized Controlled Trial Number (ISRCTN): 58808893; http://www.controlled-trials.com/ISRCTN58808893 (Archived by Webcite at http://www.webcitation.org/6pmrDRnGt)

## Introduction

A recent meta-analysis showed that mindfulness-based interventions contribute to reducing depressive symptoms (*d*=0.30 at 2 months; *d*=0.23 at 3-6 months follow-up [1]) and may be beneficial in the treatment of outpatients with acute depression [2]. Although some studies evaluated mindfulness-based interventions for inpatients with psychosis or borderline personality disorder (eg, [3,4]), to our knowledge, only one study has evaluated mindfulness techniques in depressed inpatients [5]. In this uncontrolled pilot study, an 8-session mindfulness program yielded significant pre-post changes in depression and mindfulness. However, attrition was considerable with only half of the participants completing the intervention.

The continuous practice of mindfulness exercises is a key component of mindfulness-based interventions. Time spent on formal mindfulness practices, such as body scan and mindfulness on breathing, is positively associated with outcome [6]. However, with a large share (38%) of people with depression who received mindfulness-based cognitive therapy (MBCT) not regularly practicing at home, lack of homework compliance is a frequent problem [7].

Mobile health (mHealth) apps might increase the effectiveness of mindfulness-based interventions by improving homework compliance. Being simple and efficient, the mobile phone short message service (SMS) is increasingly used to assist the delivery of mental health care [8]. A growing number of studies show that various apps of texting (monitoring, feedback, communication, homework reminders) contribute to improving uptake and outcome of mental health care [9,10].

Pilot studies have evaluated the use of texting to support homework assignments for people with depression receiving cognitive behavioral therapy [11], and as part of a Web-based monitoring and feedback intervention with a focus on mindfulness and acceptance [12]. Moreover, although recent studies have evaluated the effectiveness of Web-based and mobile phone apps, some of them also with a focus on mindfulness [13-18], there is a lack of research on the feasibility of texting interventions to support mindfulness practice in people with depression.

Thus, we developed a low-intensity program using texting to support postdischarge mindfulness practice in people with depressive symptoms receiving inpatient treatment. This randomized controlled pilot study investigated (1) participation rates during the different stages of the study and the intervention (recruitment, introductory group, randomization, texting, mindfulness exercises during follow-up, return rates of follow-up questionnaires); (2) satisfaction with the mindfulness training and the text message intervention; and (3) feasibility of the outcome measures.

## Methods

### Design

Recruitment for the study “An SMS-Assisted Mindfulness-based Intervention for Relapse Prevention in Depression” (MIND-S, ISRCTN58808893) took place between September 2013 and June 2014 at Ulm University’s Department of Psychiatry II in Günzburg, Germany. The hospital provides acute and long-term inpatient mental health care for a catchment area of about 671,000 inhabitants in rural Bavaria.

MIND-S was a pilot two-arm randomized clinical trial. Participants were invited to attend mindfulness group sessions at the hospital before discharge. Randomization took place shortly before discharge, with participants allocated to the intervention group receiving a simple texting intervention to support mindfulness exercises at home. Data was collected at three measurement points: (1) baseline (after giving informed consent, before or shortly after the first mindfulness group session), (2) prerandomization (shortly before discharge), and (3) follow-up (4 months after discharge). The study was approved by Ulm University’s Ethics Committee.

### Participants

Participants were included if they were inpatients or day patients aged 18-75 years, and showed symptoms of depression according to the clinical judgment of their inpatient therapist. Exclusion criteria were the presence of psychotic symptoms or a history of schizophrenia, current manic state, risk of a dissociative crisis, severe cognitive impairment, persistent severe substance abuse, suicidality or risk of self-harm during the current illness episode, insufficient command of the German language, and lack of a mobile phone. To identify eligible patients, therapists were informed about the study and the hospital database was regularly monitored. Therapists of possibly eligible patients were asked to give a more detailed account regarding inclusion and exclusion criteria. Eligible patients were contacted personally by the first author SK and provided with oral and written information about the study, asked to give informed consent and to complete the baseline questionnaire, and invited to attend the introductory mindfulness group. As the mindfulness group was open to all inpatients, several participants asked to visit the group once, before they gave their informed consent, and completed the baseline questionnaire shortly (maximum 1 day) afterwards.

### Randomization

After participants returned the prerandomization questionnaire, they were randomly allocated at a 1:1 ratio to the intervention or the control group. Randomization was based on a centralized procedure, which was coordinated by the Heidelberg site independent of the recruiting clinical site (Günzburg) using computer-generated random numbers.

### Intervention

#### Part 1: Mindfulness Group Training During Inpatient Treatment

The mindfulness instructions used in this study were based on the exercises suggested by Segal and colleagues [19] on mindful breathing, mindful walking, and the “body scan.” This program (“MBCT”) was originally developed for formerly depressed outpatients. Since participants were acutely depressed, all exercises were shortened to a maximum duration of 10 min and guided throughout. Following a general introduction on the principles of mindfulness and self-compassion in the context of depression, each of the following guided exercises was practiced in the group for 5-10 min: (1) mindful breathing, (2) mindful walking, and (3) mindfulness of the body (“body scan,” with a focus on feet and legs). Each participant received a short written description of the exercises to take home after discharge from hospital, and it was recommended to practice one or more of the exercises about once a day for at least 5 min.

The mindfulness group training was provided by the first author, who is a licensed cognitive behavioral psychotherapist with 7 years of experience in both inpatient group therapy and mindfulness practice, and who completed introductory courses in mindfulness-based treatments (but did not receive a full training in mindfulness-based stress reduction or MBCT). The 60-min sessions took place weekly, with the manualized contents described above being repeated every time. New participants could join the group at any time. The group was offered as a part of the standard clinical treatment and was open also to patients who were not participating in the study. However, therapists were asked to only send patients who match the study criteria. Study participants were required to attend at least once in order to continue the study.

#### Part 2: Texting After Hospital Discharge

Participants allocated to the intervention group were asked to send a text message via their mobile phones to the study center whenever they practiced one or more of the mindfulness exercises. The text message should contain information on kind (A for “Atmen,” ie, mindful breathing; G for “Gehen,” ie, mindful walking; K for “Körper;” ie, mindfulness of the body) and duration of the exercise (minutes exercised), resulting in a short alphanumeric code (eg, A10 for a 10-min practice of mindful breathing, or G5K5 for 5 min of mindful walking followed by a 5-min body scan). As a reminder, each participant received a brief pocket guide explaining the code translations to be sent via text message. After sending a text message, the participant received an automated reply, which consisted of (1) a brief positive reinforcing feedback that was drawn from a pool of 86 messages (eg, “Great! Try to be kind to yourself while practicing.”), which were formulated in advance by the study team based on the MBCT literature and randomly assigned by a computer program; and (2) the total time (in minutes) the participant had practiced since the beginning of the text message intervention (eg, “You have already practiced 25 min so far”). If a participant’s text message did not match the required format, he or she received a message on how to use the program. A reminder was sent if no text messages were received for more than 1 week. Reminders were continuously sent every week until the end of the intervention in case of persistent nonresponse. We decided to send reminders weekly instead of daily because the main function of the messages was to gently reinforce training behavior (self-management) rather than merely reminding patients to practice the mindfulness exercises. SK introduced participants to the text message intervention, which started immediately after discharge from hospital and lasted 4 months. The few participants with no texting experience received instructions on how to send and receive text messages. The intervention manual is available from the authors upon request.

Participants of the control group attended the mindfulness training group and were asked to regularly practice the exercises at home. They did not receive text message assistance during follow-up. The intervention was an add-on to treatment as usual. There were no constraints for participants to utilize any other treatment during the study period. All participants received €25 after returning the follow-up questionnaire. Additionally, participants in the intervention group received €10 at hospital discharge to cover their costs for sending text messages.

### Measures

Outcomes of the intervention to be expected include broader effects on depression (severity of depressive symptoms and perseverative thinking) and on aspects more closely related to the nature of the intervention (mindfulness and self-compassion).

*Severity of depressive symptoms* was measured with the German version of the Brief Patient Health Questionnaire-9 Item (PHQ-9 [20]), which assesses the DSM-IV criteria for major depression via patient self-report with 9 items on a 4-point Likert scale ranging from 0 (“not at all”) to 3 (“nearly every day”). The sum score ranges from 0 to 27 with higher scores indicating more severe depression.

The German version of the Perseverative Thinking Questionnaire (PTQ [21]) assesses *repetitive negative thinking* without referring to depressive symptoms in the item formulation. This self-report questionnaire consists of 15 items answered on a 5-point Likert scale ranging from 0 (“never”) to 4 (“almost always”), which describe how participants typically think about negative experiences or problems (eg, “My thoughts repeat themselves”). The total score ranges from 0 to 60, with higher scores indicating more repetitive thinking.

*Mindfulness* was assessed with the 14-item short form of the Freiburg Mindfulness Inventory (FMI; German: “Freiburger Fragebogen zur Achtsamkeit,” FFA-14 [22]). Items are rated on a 4-point Likert scale ranging from 1 (“rarely”) to 4 (“almost always”). The sum score ranging from 14 to 56 was used, with higher scores indicating more mindfulness.

*Self-compassion* was assessed with the German version (SCS-D [23]) of the short form of the Self-Compassion Scale (SCS-SF [24]). The 12 items (eg, “I’m disapproving and judgmental about my own flaws and inadequacies”) are rated on 5-point Likert scale ranging from 1 (“very rarely”) to 5 (“very often”). Higher mean total scores indicate more self-compassion.

A number of instruments measured feasibility and acceptance of the intervention at the various stages of the trial. First, *mindfulness practice* was measured via a short self-constructed questionnaire at follow-up. Participants were asked how often they practiced each of the three mindfulness exercises during each of the 4 months of the intervention period, yielding total number of exercises. Also at follow-up, participants were asked how long on average they practiced each of the three exercises in each of the 4 months. The mean duration per exercise was multiplied with the number of times this exercise was practiced in a given month, and this information was summarized for the three exercises over 4 months, yielding total duration of home practice per exercise. If participants reported an exercise without specifying the time spent on exercising, missing data was replaced by the grand means based on available data for each type of exercise (9 min for mindfulness breathing, 11 min for mindfulness walking, and 14 min for mindfulness of the body). Extreme outliers were omitted (3 participants in the control group who reported to have practiced over 200 times). Second, at baseline *, willingness to send text messages* (“Do you think you would send a text message after practicing?”) and *previous experience with the texting and mindfulness exercises* were assessed with 1-item questions each. Third, *satisfaction with the text message–based intervention* was assessed at follow-up with a 20-item adaptation of a questionnaire developed and used in earlier research of our group [25], asking on a 5-point rating scale (“I do not agree,” “I rather not agree,” “I somewhat agree,” “I fairly agree,” and “I totally agree”) for general satisfaction, satisfaction with the text message feedback, and technical problems. Fourth, *satisfaction with the mindfulness intervention* was assessed via a self-constructed 10-item questionnaire at discharge and follow-up, asking on the same 5-point rating scale about satisfaction with the mindfulness introduction, as well as about potential problems with and subjective effects of the mindfulness exercises. Fifth, at follow-up, participants of both groups were asked to rate the *usefulness* of the intervention as a whole (“yes, it helped me a lot,” “yes, it helped me somewhat,” “it neither helped nor harmed me,” “no, it rather harmed me,” and “no, it harmed me a lot”), including the option to add open-ended comments and suggestions.

*Sociodemographic information* was assessed at baseline. Furthermore, inpatient therapists were asked to document the *diagnoses* of their participating patients at study intake according to ICD-10.

### Data Analysis

According to recommendations for reporting results of pilot studies [26,27], reporting of results focuses on descriptive statistics, that is, mean and standard deviation for continuous variables, and frequencies and percentages for categorical variables. Feasibility indicators include numbers of patients eligible, willing to participate in the study, and to be randomized; numbers of participants lost to follow-up; intended, and actual use of the text message intervention; the number of text messages sent during the intervention; as well as frequency and duration of home practice.

Chi-square and *t*-tests were used to analyze differences of feasibility indicators by allocation. For the total scores of PHQ-9, PTQ, FMI, and SCS-D group by time interaction effects were tested using repeated measures analysis of variance. Additionally, effect sizes *d* (standardized mean between-group differences corrected for prerandomization differences) were calculated for all outcome measures.

## Results

### Sample

Socioeconomic status was assessed at baseline (see Table 1). Participants were 44-years-old on average, most of them were female, married, or living together with a partner (see Table 1). Level of education was predominantly low, and most were working at least part-time. Mean duration since the first occurrence of depressive symptoms was 11 years. According to their PHQ-9 score at baseline, more than three quarters (81.1%) of the patients showed substantial symptoms of depression (PHQ-9 ≥10). At baseline, participants who were later allocated to the intervention group were less depressed and showed more self-compassion than participants of the control group. The majority of the participants had at least some experience with texting. Regarding mindfulness exercises, about half had tried mindfulness practice once, a few were practicing regularly for up to 5 years.

### Participation in the Introductory Group and the Study

**Figure 1:**
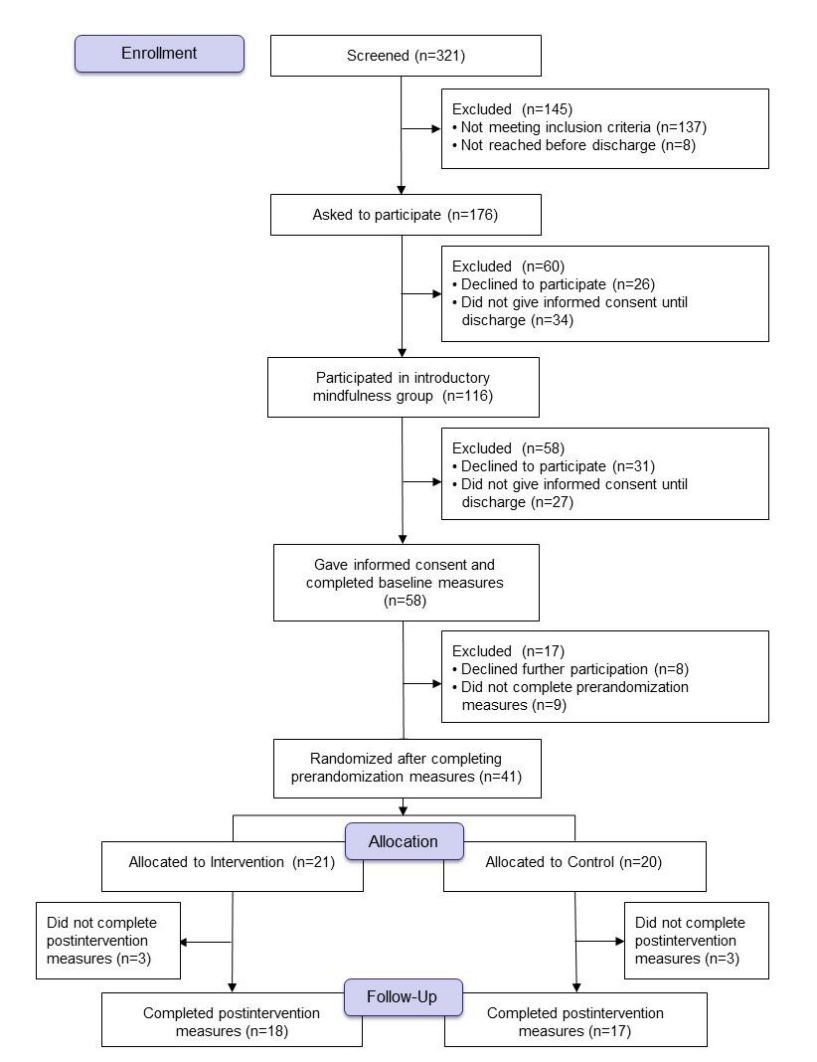
Participant flow through the stages of the trial.

It was found that 54.8% (176/321) of the screened patients were judged as eligible and invited to participate in the study. Of these, 65.9% (116/176) visited the introductory group at least once. The mean number of group visits of all participants (later randomized or not) was 2.4 (SD 1.8, range 1-9), the number of group attendees varied between 1 and 19 (mean 6.9, SD 3.5). In total, 33.0% (58/176) of the invited patients consented to participate in the study and completed baseline measures. Of the latter, 71% (41/58) completed prerandomization measures and were randomized. Randomized participants took part in the introductory group 3.4 times (SD 2.0) on average (intervention: mean 3.3, SD 2.0; control: mean 3.5, SD 2.1). Follow-up data were available for 85% (35/41) of the randomized participants, with no differences by allocation. Figure 1 depicts the (simplified) flow of participants through the stages of the trial.

Most common reasons to decline study participation (not systematically assessed) were not wanting to complete questionnaires (5%; 3/57) or using the mobile phone (4%; 2/57), a lack of interest in mindfulness practice (5%; 3/57), and feeling that participation in the study would be “too much” (5%; 3/57). Most patients (70%; 40/57) did not give any reason. Still, of the 32.4% (57/176) of patients who were not interested in study participation, 54% (31/57) attended the mindfulness training group at least once.

**Table 1:** Characteristics of study participants.

Characteristics		IG^a^ (n=21)	CG^b^ (n=20)	Differences
Age (in years), mean (SD^c^)		43.4 (12.7)	44.5 (13.5)	t_39_=−0.26; *P*=.80
Gender (female), n (%)		13 (61.9)	15 (75.0)	χ^2^_1_=0.8; *P*=.37
**Level of education**				
	Qualification for university entrance, n (%)	3 (14.3)	4 (20.0)	χ^2^_1_=0.2; *P*=.63
	Lower qualification, n (%)	18 (85.7)	16 (80.0)	
**Marital status**				
	Married or living together with partner, n (%)	10 (47.6)	11 (45.0)	χ²_2_=0.4; *P*=.84
	Single, n (%)	7 (33.3)	5 (25.0)	
	Other, n (%)	4 (19.0)	4 (20.0)	
**Employment status**				
	Full-time, n (%)	8 (38.1)	6 (30.0)	χ²_3_=0.5; *P*=.93
	Part-time, n (%)	5 (23.8)	5 (25.0)	
	Unemployed, n (%)	6 (28.6)	6 (30.0)	
	Other, n (%)	2 (9.5)	3 (15.0)	
**Primary diagnosis**				
	Major depression^d^, n (%)	16 (76.2)	19 (95.0)	χ^2^_1_=2.9; *P*=.09
	Other^e^, n (%)	5 (23.8)	1 (5.0)	
Illness duration (years), mean (SD)		10.8 (12.2)	11.7 (8.4)	t_38_=−0.29; *P*=.78
PHQ-9^f^ (depressive symptoms), mean (SD)		12.74 (5.69)	18.61 (4.86)	t_35_=−3.37; *P*=.002
PTQ^g^ (perseverative thinking), mean (SD)		38.50 (10.97)	45.32 (9.95)	t_35_=−1.98; *P*=.06
FMI^h^ (mindfulness), mean (SD)		30.56 (5.53)	27.16 (4.91)	t_35_=1.98; *P*=.06
SCS-D^i^ (self-compassion), mean (SD)		2.53 (0.76)	2.05 (0.59)	t_36_=2.15; *P*=.039
**Experience with mindfulness exercises**				
	None, n (%)	7 (35.0)	10 (50.0)	χ²_2_=1.7; *P*=.44
	Tried once, n (%)	12 (60.0)	8 (40.0)	
	Experienced, n (%)	1 (5.0)	2 (10.0)	
**Experience with texting**				
	None n (%)	5 (23.8)	3 (15.0)	χ²_2_=3.2; *P*=.20
	Some experience, n (%)	4 (19.0)	9 (45.0)	
	Very experienced, n (%)	12 (57.1)	8 (40.0)	

^a^IG: intervention group.

^b^CG: control group.

^c^SD: standard deviation.

^d^ICD-10=F32.1, F32.2, F33.1, or F33.2.

^e^ICD-10=F31.4, F40.01, F41.0, F43.2, or F61.0.

^f^PHQ-9: Patient Health Questionnaire.

^g^PTQ: Perseverative Thinking Questionnaire.

^h^FMI: Freiburg Mindfulness Inventory.

^i^SCS-D: Self-Compassion Scale; Missing values: illness duration: N=1 (IG), PHQ-9: N=2 (each IG and CG); PTQ, FMI: N=3 (IG), N=1 (CG); SCS-D: N=2 (IG), N=1 (CG).

### Participation in the Text Message Feedback Intervention

Before randomization, 59% (24/41) of the study participants expressed their intent to send text messages after practicing a mindfulness exercise if randomized into the intervention group. It was found that 20% (8/41) of the participants indicated that they would not and 22% (9/41) stated that they would “rather not” use the text message assistance. Of the 24 participants expressing intent to send text messages, 50% (12/24) were later assigned to the intervention group. At follow-up, about two-third of the participants in the intervention group reported that they had not (18%) or not always (47%) sent a text message after mindfulness practice, whereas about one-third reported that they had done so always (12%) or most of the time (24%).

During the intervention period, participants sent 294 text messages reporting 395 exercises. Of the 21 participants, 81% (17/21) in the intervention group sent at least one text message and 67% (14/21) of participants texted more than once. Two participants sent at least one message a week. On average, participants sent 14.00 (SD 21.00, median 9, range 0 *-* 91) messages, reporting 18.81 (SD 5.44, median 12) exercises during 4 months, that is, about one exercise per week. Correlations of the number of text messages with several parameters are reported elsewhere [28]. The mean duration per exercise reported via text message was 9.94 min (SD 5.38) with mindfulness of the body exercises showing the longest duration (mean 13.84, SD 4.13, N=103), followed by mindful breathing (mean 8.95, SD 5.48, N=211), and mindful walking (mean 7.54, SD 3.70, N=81).

### Satisfaction with the Interventions

**Figure 2:**
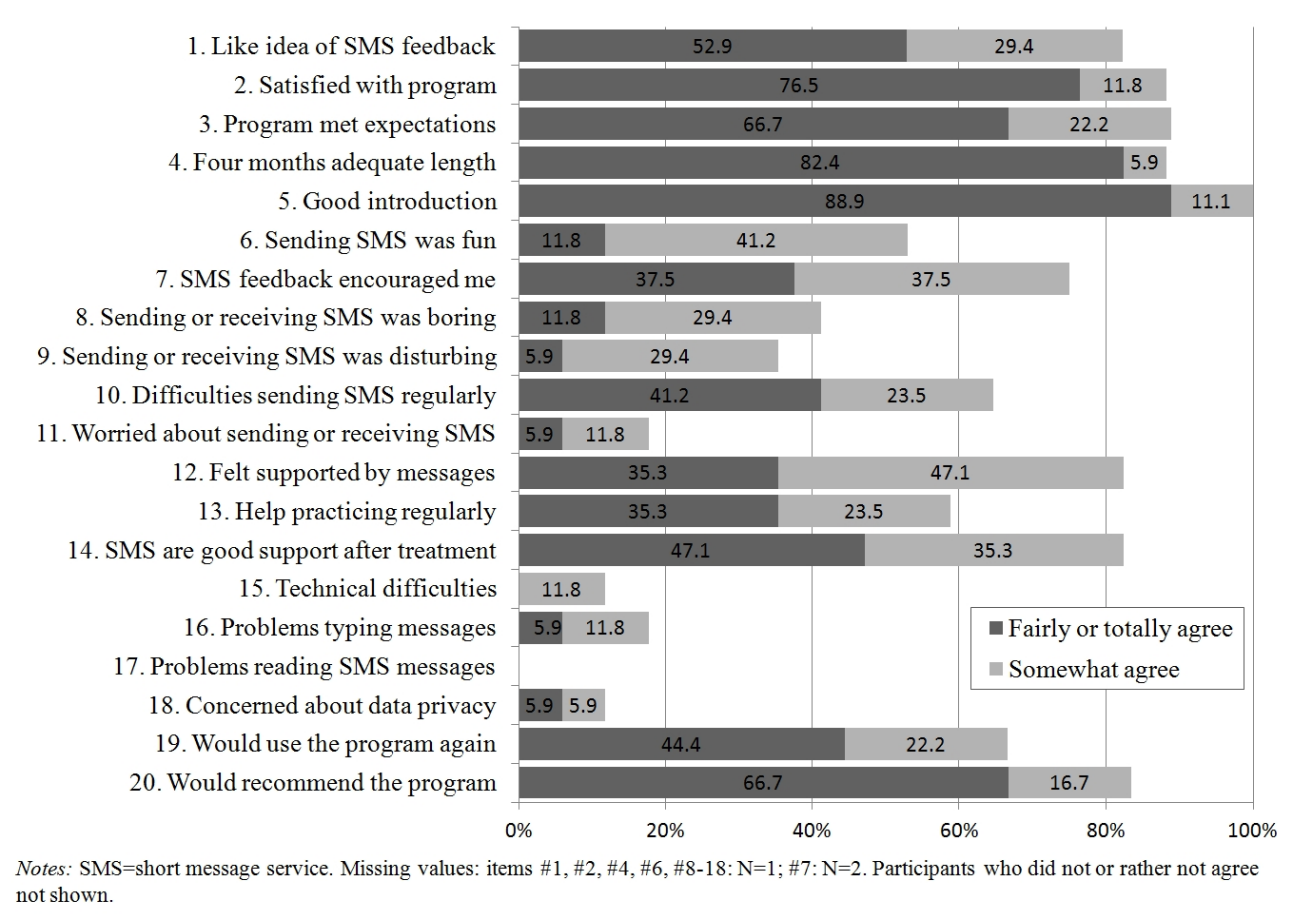
Satisfaction with the text message intervention.

Findings about satisfaction with the text message intervention are shown in Figure 2. Overall, participants showed high satisfaction. Specifically, the program met the expectations of most of the participants, more than half reported that it helped them to practice the mindfulness exercises regularly, and most felt generally supported by the text message feedback. Two-third reported that they would use the program again, and the majority indicated that they would recommend it to a friend. However, about two-third of the participants stated that they found it difficult to send messages on a regular basis, and some were concerned about data privacy.

**Figure 3:**
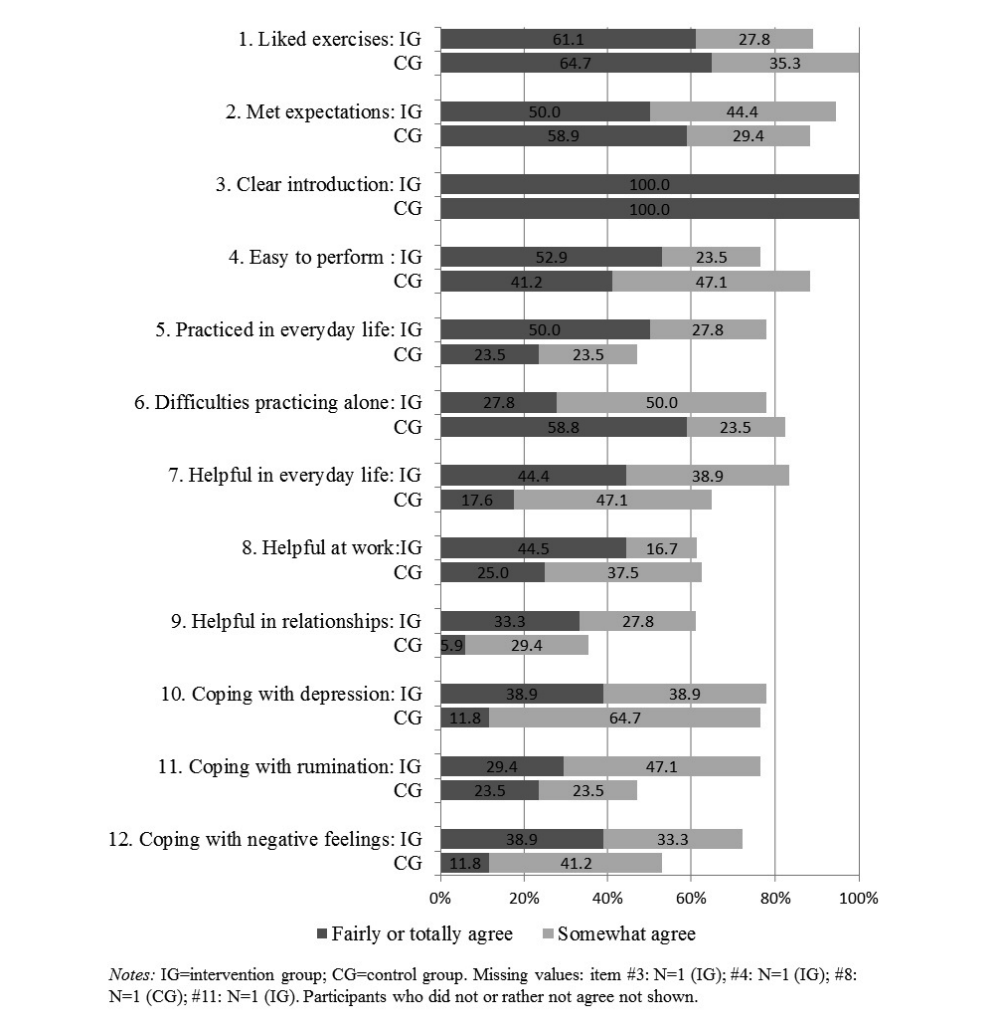
Satisfaction with the mindfulness introduction and exercises at follow-up.

At follow-up, participants of both groups were asked to evaluate the mindfulness exercises. The introduction into the mindfulness exercises at the hospital appeared appropriate and clear to all (see Figure 3). While the majority of the intervention group found that they were able to practice mindfulness in everyday life, this was the case for less than half of the participants of the control group. Furthermore, in comparison to the control group, more participants in the intervention group perceived the mindfulness exercises helpful in relationships and in coping with rumination and negative feelings. Participants in the control group reported more difficulties in practicing the mindfulness exercises alone.

At follow-up, 67% (35/53) of the participants in both intervention and control groups reported that taking part in the study helped them to some degree, the rest said it neither helped nor harmed them. The rate of agreement was higher in the intervention group (78%) than in the control group (53%). Of the 11 participants who provided written feedback at follow-up, 2 stated that they often forgot to send a text message, another 2 wrote that the weekly text message reminders were perceived as helpful, and 1 recommended further reminders in larger intervals. One participant stated that practicing in the group was easier than alone, and another one suggested to provide audio recordings of the exercises, and finally one participant recommended to offer the inpatient group sessions more frequently.

**Table 2:** Effect of the intervention on mindfulness practice and outcomes (N=35).

Outcome measures	Prerandomization		4-month follow-up		Test statistics	*P* value	Effect size (95% CI)
	IG^a^, mean (SD^b^)	CG^c^, mean (SD)	IG, mean (SD)	CG, mean (SD)			
Number of exercises practiced	7.88 (4.92)	10.06 (6.80)	62.67 (61.64)	48.71 (44.28)	*t*_30_=−0.72	.48	0.25 (−0.45 to 0.96)
Total time practiced (min)	23.75 (13.11)	35.13 (16.55)	642.17 (815.26)	579.50 (784.28)	*t*_30_=−0.22	.83	0.08 (−0.62 to 0.78)
PHQ-9^d^ (depressive symptoms)	37.47 (6.49)	32.00 (6.83)	8.94 (6.61)	12.06 (7.24)	*F*_1,31_=0.17	.68	0.14 (−0.53 to 0.82)
PTQ^e^ (perseverative thinking)	3.10 (0.77)	2.61 (0.85)	26.25 (19.28)	33.19 (16.33)	*F*_1,30_=0.85	.37	−0.26 (−0.84 to 0.31)
FMI^f^ (mindfulness)			36.40 (8.72)	32.53 (6.40)	*F*_1,28_=0.44	.51	0.25 (−0.49 to 0.99)
SCS-D^g^ (self-compassion)			3.11 (1.06)	2.63 (0.71)	*F*_1,29_=0.01	.94	0.02 (−0.59 to 0.63)

^a^IG: intervention group.

^b^SD: standard deviation.

^c^CG: control group.

^d^PHQ-9: Patient Health Questionnaire-9 Item.

^e^PTQ: Perseverative Thinking Questionnaire.

^f^FMI: Freiburg Mindfulness Inventory.

^g^SCS-D: Self-Compassion Scale; Missing values: PHQ-9: N=1 (each IG and CG); PTQ: N=2 (IG), N=1 (CG); FMI: N=3 (IG), N=2 (CG); SCS-D: N=2 (each IG and CG).

### Feasibility of the Outcome Measures

The effects of the post-hoc assessment of mindfulness home practice (number and duration of exercises practiced) and the four selected outcome questionnaires (PHQ-9, PTQ, FFA, and SCS-D) yielded near zero to small, nonsignificant effect sizes (see Table 2). Note that, due to different scoring, a positive effect size on the symptom measures (PTQ, PHQ) indicates an outcome in favor of the intervention group, whereas on the FMI and the SCS-D, a positive effect size indicates a result in favor of the control group.

The largest but still small differences were found on perseverative thinking, self-reported mindfulness, and the number of reported exercises. Effects for the former two measures were against expectations because the control group scored lower on perseverative thinking (*d*=−0.26) and higher on mindfulness (*d*=0.25) than the intervention group. The number of exercises practiced tended to be higher in the intervention group (*d*=0.25), and there was small effect on the PHQ-9 in favor of the intervention group.

## Discussion

### Principal Findings

To our knowledge, this is the first study to evaluate the feasibility of a simple, low-intensity texting app to support mindfulness practice in people with depressive symptoms after inpatient psychiatric treatment. The study showed that a considerable proportion of people with depressive symptoms receiving in inpatient treatment are interested in a mHealth mindfulness program, and that most participants were satisfied and experienced the intervention as helpful.

### Participation in the Introductory Group and the Study

According to introductory group participation rates, the mindfulness exercises seemed to interest about two-third of the target population of psychiatric inpatients with depressive symptoms. Only half of them could be motivated to participate in the study (eg, fill out questionnaires and participate in the texting intervention). However, once they decided to participate in the study, most patients stayed in the trial until the end.

Retention rates were 71% for the first (prerandomization) and 85% for the second (postrandomization) part of the study, falling within the upper range of other mHealth studies (43-100% [29]). Similar, sometimes higher, attrition rates were found in studies evaluating mindfulness-based interventions in depressed patients delivered face-to-face (49% [5]; 8-38% [2]) or via the Internet or mobile phone (57% [14]; 38% [15]). Good retention rates speak to the feasibility of the study design and committed study staff. However, the possibility of a selection bias toward including rather motivated and compliant patients cannot be ruled out.

### Participation in the Text Message Feedback Intervention

On average, participants sent about one text message per week which falls behind expectations, as daily practice was recommended. However, this result is not surprising, as before the beginning of the intervention, less than 60% of the randomized participants indicated that they intended to text. Comparing the postintervention self-report with the information from the text messages, participants texted only about every third time after a mindfulness exercise. Likewise, about two-third of the participants reported that they had not or not always sent a text message after they had practiced, and that they had difficulties texting regularly. However, the possibility of a combined effect of a social desirability bias and a 4-month recall bias should be taken into account. Nevertheless, participation patterns are comparable with another study which examined text-messaging support in the treatment of people with depression (65% response rate to text message reminders [11]), indicating that expectations might have been unrealistic. Another explanation could be that a considerable number of participants did not feel the need to be supported every time they practiced. Taken together, these findings suggest a need to enhance mode of delivery and content of text messages including tailoring feedback to increase subjective meaning. This might also be achieved by sending daily queries inquiring about type and duration of practice.

### Satisfaction With the Interventions

Participants of the intervention group were predominantly satisfied with the texting intervention. Most felt supported and encouraged by the messages, and over 80% would further recommend the program. Although use of the texting intervention was moderate, in absolute values, considerably more patients in the intervention than in the control group perceived the mindfulness exercises as helpful, especially regarding coping with rumination and negative feelings. More patients in the intervention group than in the control group stated that they had practiced in daily life. Furthermore, compared with the control group, in the intervention group, fewer patients at least fairly agreed that they had problems practicing alone. Although these are no significant results and should be regarded with caution, they might indicate that the text message feedback supports mindfulness home practice and could increase the effects of mindfulness exercises as intended [30].

### Feasibility of the Outcome Measures

Although the main outcome measures (amount of home practice, depressive symptomatology) depict a small effect of the intervention, the additional measures (rumination, mindfulness, self-compassion) yielded zero or small effects in favor of the control group. Due to the small sample size, no final conclusion can be drawn. However, these results indicate that either the questionnaires were insensitive to changes, the dosage of intervention was not sufficient, or the observation period was too short to detect changes. It could be hypothesized that mindfulness and self-compassion are rather trait-like, less susceptible to change, and thus, need a longer and more intense intervention to change.

### Limitations

This study has a number of limitations. First, the introduction to the mindfulness exercises and the text message intervention was provided by the first author, who also was involved in data collection and analysis. Second, the intervention was only implemented at one hospital, suggesting limited generalizability of findings. Third, there could have been a selection bias, as the inpatient therapists could recommend a patient to participate or not to participate in the study due to their subjective judgment. Fourth, the mindfulness introduction was a shortened and adopted version of other mindfulness-based programs, and has not been validated in this population. Fifth, there might be a recall bias regarding the post hoc assessment of the mindfulness exercises practiced in the follow-up period, combined with a social desirability bias. There might have been a tendency to over-report the mindfulness practice in the intervention group. Finally, due to the small sample size, all results of this pilot study should be interpreted with caution.

### Conclusions

Taken together, the positive evaluations of the MIND-S program by the participants indicate that, in general, mindfulness practice augmented by text message feedback is a feasible intervention. However, the moderate use of the texting intervention and the mixed effects imply that dose and ingredients of the intervention should be increased for this group of patients in a future, full-scale RCT. Additional components might include expert or peer support such as regular mindfulness group visits after discharge, audio or video material, more frequent reminders, or individual expert support via texting, email or chat-groups. To minimize recall bias, frequency and duration of mindfulness practice should be assessed more frequently during the follow-up period. Furthermore, the intervention should also be tested in other populations (such as previously depressed outpatients) to assess the differential indication. A larger study will also allow to investigate the effects of the intervention, as well as moderators of the effect of mindfulness practice and text message feedback on clinical outcome.

## Abbreviations

FMI: Freiburg Mindfulness Inventory
MBCT: mindfulness-based cognitive therapy
mHealth: mobile health
PHQ-9: Patient Health Questionnaire-9
PTQ: Perseverative Thinking Questionnaire
SCS: Self-Compassion Scale
SMS: short message service
